# Mobile Diagnostic Clinics

**DOI:** 10.1021/acssensors.4c00636

**Published:** 2024-05-22

**Authors:** Roni Baron, Hossam Haick

**Affiliations:** † Department of Biomedical Engineering, Technion—Israel Institute of Technology, Haifa 3200003, Israel; ‡ Department of Chemical Engineering and the Russell Berrie Nanotechnology Institute, Technion—Israel Institute of Technology, Haifa 3200003, Israel

**Keywords:** healthcare, clinics, sensors, wearable, diagnosis, remote management, real-time, continual monitoring

## Abstract

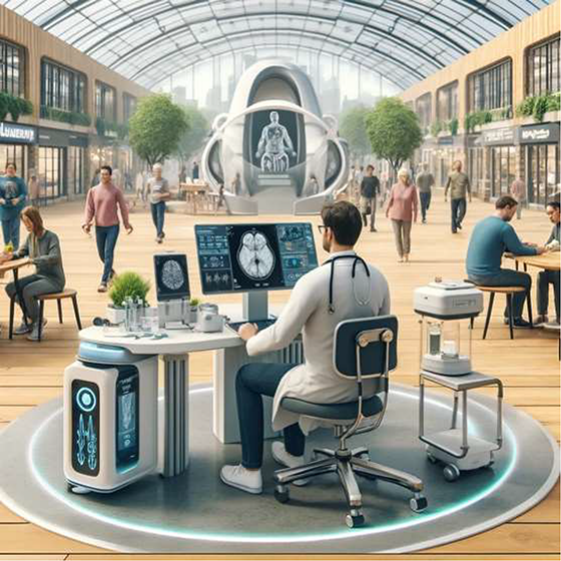

This article reviews the revolutionary impact of emerging technologies and artificial intelligence (AI) in reshaping modern healthcare systems, with a particular focus on the implementation of mobile diagnostic clinics. It presents an insightful analysis of the current healthcare challenges, including the shortage of healthcare workers, financial constraints, and the limitations of traditional clinics in continual patient monitoring. The concept of “Mobile Diagnostic Clinics” is introduced as a transformative approach where healthcare delivery is made accessible through the incorporation of advanced technologies. This approach is a response to the impending shortfall of medical professionals and the financial and operational burdens conventional clinics face. The proposed mobile diagnostic clinics utilize digital health tools and AI to provide a wide range of services, from everyday screenings to diagnosis and continual monitoring, facilitating remote and personalized care. The article delves into the potential of nanotechnology in diagnostics, AI’s role in enhancing predictive analytics, diagnostic accuracy, and the customization of care. Furthermore, the article discusses the importance of continual, noninvasive monitoring technologies for early disease detection and the role of clinical decision support systems (CDSSs) in personalizing treatment guidance. It also addresses the challenges and ethical concerns of implementing these advanced technologies, including data privacy, integration with existing healthcare infrastructure, and the need for transparent and bias-free AI systems.

## Overview of Current Healthcare Systems

The role of diagnostic clinics as cornerstones in modern healthcare systems is unquestionable. Whether operating as separate diagnostic centers or as sections within a hospital, these entities have become the first point of contact for patients seeking medical care that covers a broad range of services, from regular medical check-ups and tests to more complicated emergency procedures.^1^ Nevertheless, diagnostic clinics are not without some considerable drawbacks. The dependence on experienced medical practitioners for clinical decision-making generates a bottleneck in patient treatment, restricting the number of patients that can be attended to at any given time. The World Health Organization (WHO) projects that by 2030, there will be a deficiency of 18 million health workers, which, paired with the current substantial dearth of healthcare workers, could lead to longer wait times for appointments, thus compromising the quality of care.^2^ This predicament is further accentuated in developing countries where the ratio of healthcare workers to patients is already abysmal. As an example, Africa, which bears 24% of the global disease burden, has access to only 3% of healthcare workers.^3−5^

The existing diagnostic healthcare model faces a daunting financial challenge. Significant sums of money must be invested in clinics to pay for necessary medical devices and equipment, as well as operational and managerial expenses. The global medical device market in 2020 was worth $456.9 billion and is expected to experience a significant increase.^6^ Unfortunately, many diagnostic clinics are unable to provide a lasting solution to their patients’ medical issues or advance their diagnosis or treatment, even though a considerable amount of funds has been invested. The absence of persistent monitoring ability in the conventional clinical model further exacerbates the difficulties. Regular diagnostic clinics are often just a single visit and lack continual assessment of a patient’s health condition, which can lead to missed early diagnosis of diseases.

Conventional diagnostic clinics need to be revolutionized to keep up with societal and financial demands while incorporating technological advances. An innovative healthcare platform that is easily accessible to patients is needed, serving as their first point of contact with the healthcare system, while incorporating portable and miniaturized diagnostic and screening devices. This platform would reduce the burden on the overwhelmed healthcare systems by regulating the influx of patients to central healthcare providers.^7,8^ Acting as a “gate”, this platform would give a preliminary indication of a person’s medical state, helping to determine whether a person should seek expert medical attention. This would also aid in the effort for early detection of pathologies, an essential parameter in mitigating premature death.^9^

## The Need for Continual Monitoring

As the demand for early disease detection and management grows, it is crucial to incorporate continual monitoring solutions within diagnostic clinics. Noninvasive methods of collecting health data form the foundation of these solutions, enabling the seamless tracking of a patient’s health status over time. These innovative approaches are critical in fulfilling the industry’s goal for continuous health monitoring, especially in the early stages of disease development. Continual monitoring surpasses traditional periodic assessments by utilizing point-of-care testing and other forms of intermittent sampling technologies. By adjusting the frequency of these tests to match the disease’s progression rate, it is possible to create a robust and effective continual monitoring environment. This strategy ensures a dynamic and responsive approach to patient care, keeping a close eye on disease evolution even between physical check-ups. The availability and accessibility of advanced diagnostic technologies are central to making this vision a reality.

Decentralizing diagnostic processes give patients the flexibility to undergo necessary tests outside the confines of hospitals or large medical centers, reducing the initial barriers to essential health evaluations. Integrating continual monitoring into the patient care continuum significantly enhances the capacity for early disease detection. Early diagnosis is a crucial step in improving patient outcomes and can substantially alleviate the strain on healthcare systems by minimizing the need for extensive screening procedures. In the context of cancer, where early detection is vital, shifting toward continuous monitoring and early diagnostics could result in substantial financial savings, estimated at $26 billion annually in the United States alone.^10−12^ Emphasizing the screening of asymptomatic individuals stands as the most effective strategy for early disease identification, underscoring the critical role of diagnostic clinics in achieving this goal. Therefore, incorporating continual monitoring solutions within diagnostic clinics is vital to addressing the growing need for early disease detection and management.

## From Traditional to Mobile: The Evolution of Diagnostics Through Technology

The opportunities that exist through the use of emerging technologies, such as nanotechnology, artificial intelligence (AI), wearable devices, and point-of-care (POC) *testings*, are awe-inspiring in their capacity to shape the future of healthcare. Through their synergistic application, these technologies open unprecedented possibilities, from more precise, individualized care to improved early detection of diseases to enhanced therapeutics.

Nanotechnology can alter the future of healthcare by manipulating matter at the atomic or molecular level, resulting in heightened sensitivity of diagnostic instruments such as nanosensors, which can detect biomarkers at incredibly low concentrations.^13^ AI has brought forth the potential to reshape healthcare decisions through its data analysis and predictive modeling prowess. Subsets of AI, such as machine learning (ML) algorithms, can scan through large amounts of patient data, recognizing patterns that would otherwise go undetected by the human eye. Its applications have helped to increase diagnostic accuracy, predict prognosis, and formulate personalized treatment plans, along with telemedicine enabled by wearable devices and remote monitoring tools.^14−17^ Accenture reports that AI’s implementation could result in savings of up to $150 billion for the U.S. healthcare system by 2026.^18^ Additionally, POC testing devices, portable imaging equipment, and lab-on-a-chip systems have brought diagnostics closer to home, even for the most rural locations, and have been influential in advancing personalized treatments and prosthetics, due to the emergence of 3D printing in medicine.^19,20^ Examples include the creation of hearing aids,^21^ dental implants,^22^ and a range of medical equipment.

The merging of these technologies presents the opportunity to revolutionize the healthcare service industry, enabling swift and precise diagnosis which will be realized through real-time data analysis from portable devices and nanosensors powered by AI. The potential of these cutting-edge technologies promises to deliver care that is both effective and available to all. In the future of smart, miniaturized healthcare facilities, made possible by AI, nanotechnology, and other technologies, issues such as accessibility, affordability, and continual monitoring could be adequately addressed.

## Mobile Diagnostic Clinics—A Concept

Building on the transformative potential of AI,^23−25^ nanotechnology,^26,27^ and portable diagnostic tools,^28,29^ “Mobile Diagnostic Clinics (MDCs)”—a state of concept—represent a transformative innovation in healthcare, leveraging emerging technologies to bring advanced diagnostic capabilities directly to patients. By enabling real-time data analysis and leveraging minimally invasive sensors, MDCs extend the benefits of these sophisticated technologies beyond traditional healthcare settings. This mobile approach ensures that high-quality diagnostic services are widely available, addressing critical issues of accessibility, affordability, and the need for continual health monitoring. In doing so, MDCs represent a pivotal shift toward a new era of healthcare that is both effective and accessible to all.

The design of miniaturized healthcare facilities will increase patient access to healthcare, generally featuring technologies of a much smaller size than regular clinics, making it feasible to implement MDCs in various locations such as community centers, offices, and malls. Access to these facilities helps realize the vision of continually tracking a person’s health and alerting them of any anomalies that occur—overcoming accessibility restrictions inherent in traditional clinics. Using wearable gadgets and other remote patient monitoring equipment, these clinics will be able to monitor patient health metrics continually, from afar, allowing for early diagnosis of diseases and prompt interventions. Through AI and other technologies, smart clinics can decrease operational costs, mainly in the long run, making them cost-effective.^30^ This eliminates the need for a multitude of medical personnel for tedious tasks, such as appointment scheduling and data logging, as a considerable number of these can be automated. Moreover, remote consultations will drastically reduce patient expenses, such as travel fees. By providing preliminary diagnoses remotely, time and location obstacles for patients significantly decrease.

Essential components of these miniaturized healthcare facilities are digital health tools and systems fueled by AI. AI-enabled instruments permit a broad spectrum of services, from everyday screenings to diagnosis, therapeutic advices, and continual monitoring of patient health, which allows for personalized care delivery.^31^ Machine learning algorithms examine copious amounts of patient data, discovering underlying patterns and developing an understanding of patients’ health. This initial understanding can pave the way for customized treatment plans, provided by healthcare professionals based on the acquired data. Integrating nanotechnology makes it possible to create ultrasensitive nanosensors that can detect various health markers and signs of disease in their earliest stages. Not only is this process noninvasive, but it is also usually comfortable for the patient. These devices are often cheaper than their gold-standard diagnostic counterparts. Therefore, these technologies can be manufactured and distributed in remote locations, where buying and training people to use gold-standard, expensive devices is not feasible while also realizing the goal of noninvasive, continual patient monitoring. Furthermore, with the help of portable imaging machines and lab-on-a-chip systems, even complex diagnostic procedures can be performed away from traditional healthcare facilities, ensuring fast, precise, and available results, no matter where the patient may be. This takes us one massive step closer to the goal of personalized healthcare.

**Figure 1:**
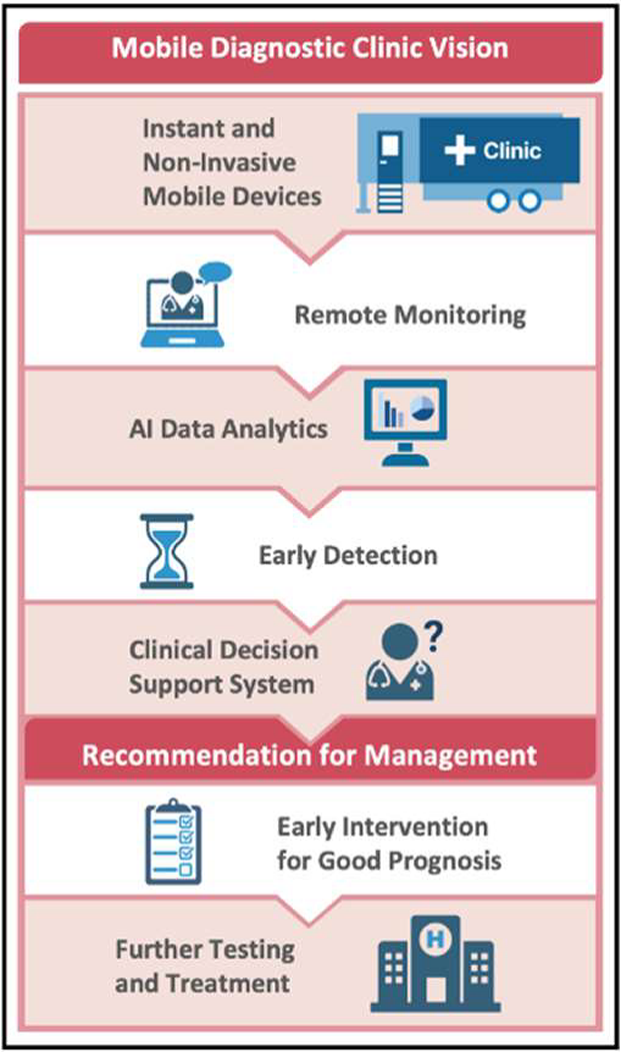
Mobile diagnostic clinics—A concept. Created with BioRender.com.

In an era dominated by AI, the evolution of healthcare through MDCs marks a significant transformation in the delivery of medical services. By integrating emerging technologies, MDCs are poised to revolutionize access to comprehensive, noninvasive health evaluations, diagnoses, and ongoing condition monitoring. ***This approach does not merely rely on multifunctional devices but emphasizes the importance of utilizing individual devices, each specialized in its function. The integration and data analysis across these devices are orchestrated by AI, providing powerful clinical decision support. This strategy leverages the precision and flexibility of these specialized tools, enabling AI to compile and interpret data for a holistic understanding of patient health.*** Consequently, this shift is expected to democratize healthcare, making it more effective, personalized, and accessible to a broader audience. The fusion of these advanced technologies within MDCs heralds a future where healthcare is seamlessly integrated into our lives, offering tailored and continual medical support that transcends traditional care models (Figure 1).

## Technological Advances for Mobile Diagnostic Clinics

Upon access to an emergency or healthcare facility, patients must undergo a series of preliminary checkups followed by more specific testing to establish a diagnosis and deciding on a treatment plan. The general workflow consists of (1) vital sign monitoring, (2) chemical workups, and (3) imaging. Below is an overview of the technologies that have been devised to aid in the incorporation of clinical diagnostics into a MDC workflow.

### Vital Sign Monitoring

It is already relatively easy for patients to monitor vital signs in the absence of a healthcare professional, in the comforts of their own homes, and as part of their daily routines. This capability is credited to the development of various small-scale sensors and portable solutions that allow for the monitoring of heart rate, blood pressure (BP), temperature, and respiratory rate.^32^ Oxygen saturation is also frequently used in the assessment of a patient’s state.^33^ The major difference between nonwearable and wearable vital sign monitoring devices lies in the sampling frequency. While wearable devices achieve continuous monitoring, nonwearable devices are usually restricted to intermittent time points.^34^

#### Portable (Non-Wearable) Devices

Most of the traditional methods for monitoring vital signs are already compatible with the MDC platform as they are small-scale and portable.

**Figure 2:**
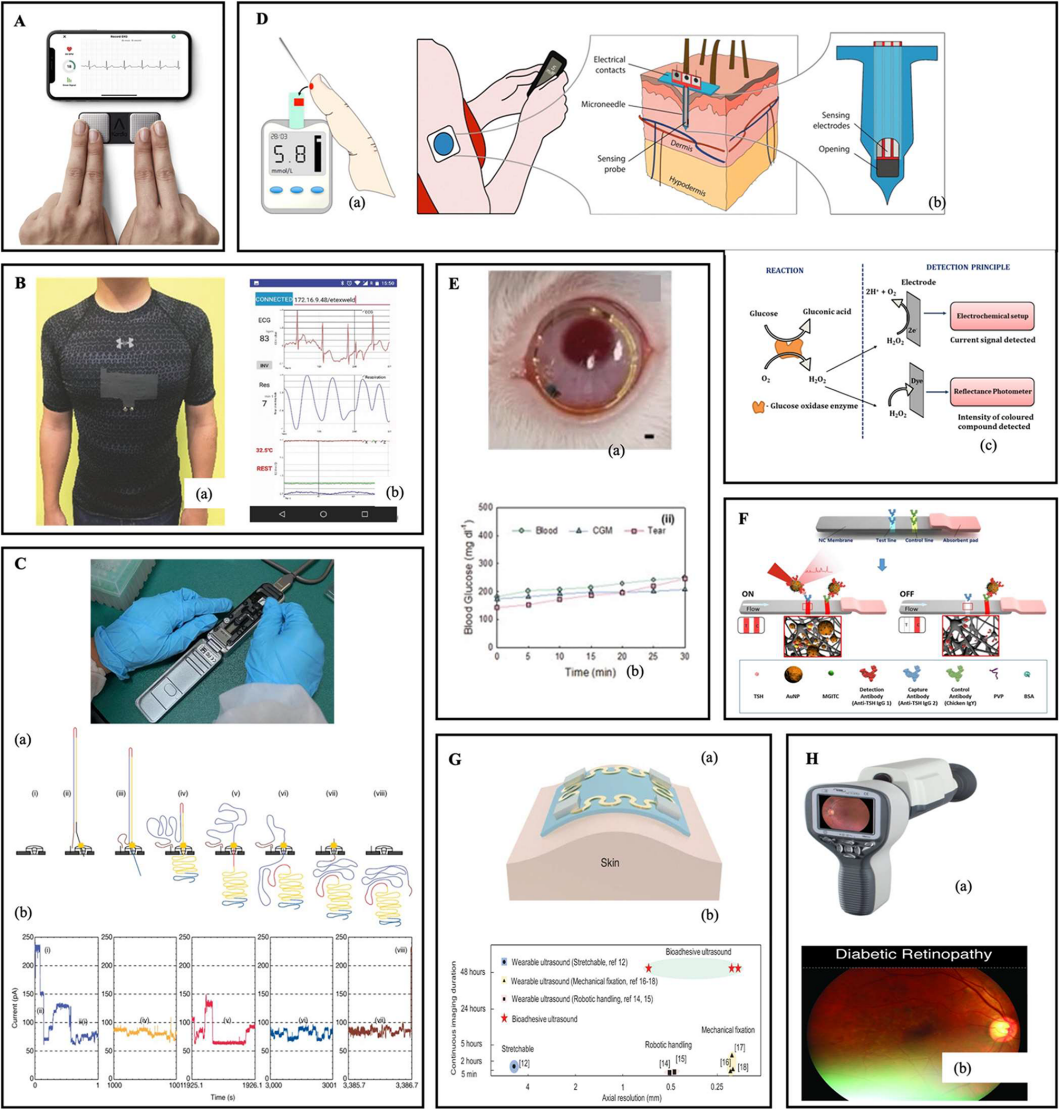
Summary of data collection devices and emerging technologies. (A) A platform to integrate smart device electrocardiogram into clinical practice. Reprinted (in part) with permission from Lambert et al. Reference^36^. Copyright 2021 Elsevier. (B) (a) Textile-based activity and ECG monitoring platform with a (b) mobile user interface. Reprinted (adapted) with permission from Tao et al. Ref (49). Copyright 2018 John Wiley and Sons. (C) (Top) MinION portable DNA sequencer. Reprinted with permission from Mongan et al. Ref (65). Copyright 2020 Springer Nature. (Bottom) Full-length read of dsDNA through the nanopore sequencer. (a) Steps in the translocation of the DNA through the nanopore. Each section of DNA is depicted by a different color (b) Raw current traces corresponding to the steps (i–viii) in (a). Each section generates a unique current trace corresponding to decipher base sequence. Reprinted (in part) with permission from Jain et al. Ref (68). Copyright 2016 BioMed Central. (D) Continuous Glucose Monitoring (CGM) Devices. (a) Finger pricking device. (b) Schematic of CGM devices. (c) Detection principles of Glucose sensors. Reprinted with permission from Kumar Das et al. Ref (72). Copyright 2022 The Electrochemical Society. (E) Bimetallic nanocatalysts in nanoporous hydrogels for CGM via contact lens. (a) In vivo CGM of smart contact lens in diabetic rabbits (scale bar: 150 μm) (b) Correlation equation between blood and tear glucose levels using a CGM with a glucometer (green), a commercial CGM (blue) and the smart contact lens (pink) for 30 min. Reprinted (in part) with permission from Kim et al. Ref (73). Copyright 2022 John Wiley and Sons. (F) Illustration of lateral flow immunoassay for detection of thyroid-stimulating hormone (TSH). Reprinted with permission from Choi et al. Ref (88). Copyright 2017 Elsevier. (G) (a) Bioadhesive ultrasound for continuous imaging. (b) Comparison of image resolutions and monitoring durations. Reprinted (in part) with permission from Wang et al. Ref (91). Copyright 2022 The American Association for the Advancement of Science. (H) (a) Pictor Plus hand-held fundus camera. Reprinted from https://www.volk.com/pages/portable-fundus-cameras. Copyright 2024 Volk Optical. (b) Diagnosis of Diabetic Retinopathy by hand-held fundus camera. Reprinted (in part) with permission from Lu et al. Ref (99). Copyright 2022 PLoS.

Heart rate can be monitored either manually, or extracted from acquired Electrocardiogram (ECG) signals. ECGs are recorded by placing three or more electrodes on the skin at predetermined locations. Devices such as KardiaMobile (AliveCor, Mountain View, CA) have emerged as Portable ECG Monitors. These gadgets collect medical-grade ECG readings that can be easily shared with healthcare professionals for a more thorough evaluation (Figure 2A). By facilitating the prompt discovery and supervision of heart issues, such as congenital heart disease and atrial fibrillation, these devices can assist in avoiding significant health complications.^35,36^

A photoplethysmogram (PPG) is obtained by measuring the changes in peripheral blood volume using an optical technique. A photodetector is placed on a patient’s fingertip or earlobe, while light is transmitted to the skin to obtain a signal.^33^ Blood volume fluctuations, as a reflection of cardiac activity, influence the absorption profile of the transmitted light.^37^ Blood oxygen saturation can be derived from PPGs.^38^

BP can be measured either using a simple sphygmomanometer, a device consisting of an inflatable cuff and a pressure gauge or with an ambulatory blood pressure (ABP) monitoring device. Common sphygmomanometers are portable, easy to use, and inexpensive, while ABPs are relatively more costly. More sophisticated sphygmomanometers integrate with a mobile app to display and classify recordings.^39^ Kurylyak et al. demonstrated that cuff-less continuous BP can be derived from the acquired PPG signals using an artificial neural network.^40^

Respiration rate is often monitored by manually counting the number of breaths a patient takes within a given time.^41^ This method is inaccurate and as such several automatic devices have been developed. These include portable respiratory monitors such as the Capnostream 35 (Medtronic, Minneapolis, MN).^42^ Respiration rate can also be extracted from PPG signals.^43^

#### Wearable Devices

Wearable technologies are embarking on a revolutionary path, moving from simply trendy accessories to essential partners in health surveillance and disease management. Today’s modern smartwatches, fitness trackers, and custom-built health sensors have been developed to monitor a range of health metrics. With the capacity to provide immediate, invaluable data, these devices enhance health outcomes and foster a personalized approach to healthcare. Smartwatches and Fitness Trackers, such as the Apple Watch (Apple, Cupertino, CA), Fitbit (Fitbit LLC, San Francisco, CA), and Garmin (Garmin Ltd., Olathe, KS), initially used for monitoring physical activity and sleep patterns, have extended their features. These devices continuously monitor important parameters like heart rate and blood pressure, pushing the notion of wellness tracking to an even higher level. Additionally, versions like the Apple Watch Series 4 have taken health tracking to a greater degree with the inclusion of ECG functions.^44,45^ Such advancements can warn users of any atypical heart rhythms that may signify severe ailments like atrial fibrillation, thereby playing a key role in early recognition and intervention.

For those with diabetes, wearable technologies have brought about innovative solutions like Continuous Glucose Monitoring (CGM) systems, including the FreeStyle Libre system (Abbott Diabetes Care, Inc., Alameda, CA). These wearables offer instantaneous glucose level readings, patterns, and warnings, simplifying diabetes management and eliminating the necessity of frequent finger-prick blood tests.^46^ Sleep Monitoring Devices are also gaining popularity in the wearable tech industry. Devices such as Fitbit Sense can record a variety of sleep health metrics, comprising total sleep time, sleep stages, and oxygen saturation levels.^47^ The knowledge obtained from this data can spotlight problems like sleep apnea, enabling users to intervene promptly and improve their sleep quality. Additionally, these devices can be used to determine respiration rates.^41^

An extra step into wearable technologies has been taken by innovation in the field of Smart Clothing. With embedded sensors and flexible printed circuit boards and electrodes, these cutting-edge garments monitor multiple health and fitness parameters. For instance, Linz et al. developed an ECG monitor embedded into a commercially available, tight-fitting T-shirt.^48^ Tao et al. developed a washable, three-textile-electrode system for the recording of ECG, temperature, and respiration rate (Figure 2B).^49^ The unification of these instances illustrates how wearable devices are altering healthcare. They have already begun delivering real-time health data, a trend that is only predicted to become more substantial with future technological advances.

### Chemical Workups

#### Point-of-Care Testing (POCT) Devices

The concept of POCT devices is transforming the medical diagnostics industry. These devices deliver laboratory-grade diagnostics directly to the patients, provide rapid, accurate, and convenient testing for various health conditions, and generate instantaneous results that help healthcare professionals make quick, informed decisions and expedite diagnosis and treatment. Here are a few examples of POCT devices: Blood Glucose Meters, such as the Accu-Chek Guide Me (Roche Diabetes Care, Inc., Switzerland), Bionime GM 110 (Bionime, Malaysia), and the Freestyle Libre, provide people with diabetes the power to monitor and manage their condition more efficiently.^46,50^ The CoaguChek XS System (Roche Diagnostics, Basel, Switzerland) is a hand-held device that enables patients on long-term anticoagulant therapies, like warfarin, to test their International Normalized Ratio levels in the comfort of their home, minimizing the need for frequent visits to the clinic and enabling timely changes to medication dosages.^51^ Cardiovascular Markers Testing is further improved with the PATHFAST (Polymedco, LLC, NY), a multiassay diagnostic system that can quickly detect conditions such as acute myocardial infarction and congestive heart failure, facilitating faster patient care decisions.^52,53^ Infectious Disease Testing has taken a giant leap with the GeneXpert System (Cepheid, Sunnyvale, CA), a device capable of performing rapid molecular tests for various diseases, including tuberculosis, HIV, and influenza strains, in under 2 h.^19,54,55^ Portable Hematology Analyzers, like the HemoCue Hb 201+ and 301 (HemoCue AB, Ängelholm, Sweden) systems, can carry out rapid and reliable hemoglobin tests to help diagnose anemia, particularly useful in rural locations.^56^ Their portability is especially useful in resource-limited settings, where access to comprehensive laboratory services may be restricted. Respiratory Monitors, such as the SpiroScout SP (Ganshorn Medizin Electronic, Niederlauer, Germany), use U/S technology to measure lung function accurately.^57^ This noninvasive, portable device delivers instant results, making it a valuable tool for people with chronic respiratory diseases, like chronic obstructive pulmonary disease and asthma. With instant diagnostics at the patient’s location, these devices improve patient outcomes and significantly boost healthcare systems’ efficiency.

#### Microfluidics and Lab-on-a-Chip (LOC) Systems

Microfluidics and LOC systems signify a fantastic breakthrough in the realm of diagnostics and biomedical research. These advanced technologies enable the manipulation of microliter-scale volumes of fluids, yielding lightweight and highly effective diagnostic solutions. One of the applications of this technology is Compact Immunoanalyzers; these devices draw on the power of microfluidics and LOC technology to carry out complete blood tests, liver function analyses, and immunoassays with merely a drop of blood.^58^ Devices such as PATHFAST have also been tested in an ICU setting for the assessment of presepsin concentration, a diagnostic marker in sepsis.^58,59^ Such devices bring accurate results in under 20 min, simultaneously analyzing multiple samples and requiring only 100 μL sample volumes, completely transforming POC diagnostics and therapeutic decision-making.

Another breakthrough technology is Portable Bioanalyzers, which use LOC systems to conduct electrophoresis on a credit-card-sized chip. Providing qualitative and quantitative analysis of DNA, RNA, and proteins in just half an hour, these analyzers present a dependable and much quicker alternative to traditional, lengthy gel electrophoresis methods.^60^ Enabled by microfluidics, Hand-held Blood Analyzers can perform a variety of tests, from blood gases and chemistries to coagulation and cardiac markers, all while offering lab-level results in mere minutes, facilitating timely treatment decisions.^61−63^ Moreover, high-throughput Polymerase Chain Reaction (PCR) systems represent ground-breaking progress in this domain. Thanks to microfluidic technology, these systems execute gene expression analysis, genotyping, and digital PCR on one chip, permitting the simultaneous handling of multiple samples and assays while guaranteeing accuracy and scalability in genetic analysis.^64^ Additionally, portable DNA analyzers allow for quick DNA testing on the go. By integrating microfluidic and nanopore technology, these systems can detect infectious diseases, pharmacogenetic conditions, and even waterborne pathogens within an hour.^65−67^ Currently, the most renowned DNA analyzer is the MinION (Oxford Nanopore Technologies, Oxford, U.K.) (Figure 2C).^68^ Lastly, compact chemistry analyzers represent miniature devices that can perform a broad range of biochemical tests, delivering results in minutes.^69^ Owing to their condensed size and user-friendly interface, they capture the concept of POC testing, potentially making diagnostics available anywhere. These instances demonstrate the boundless potential of microfluidics and LOC systems in speeding up, increasing access to, and optimizing diagnostics. The ultimate promise of these technologies lies in the possibility of delivering advanced diagnostic capabilities to underserved regions, promoting healthcare equity and accessibility. This is entirely compatible with the idea of MDCs, driving us closer to a future where every individual has access to quality healthcare, no matter their location.

#### Nanotechnology-based Devices

Nanotechnology is a driving force in revolutionizing healthcare, signaling the emergence of precision medicine. The utilization of nanotechnology to produce nanosensors gives the potential to identify disease biomarkers in their early stages. This has brought forth a new period of exact, individualized medical care. Several noteworthy nanotechnology applications bolster this idea: Nanobiosensors, the pacesetters of nanodiagnostics, use nanotechnology to detect biomarkers, such as DNA/RNA fragments, proteins, and organic molecules.^13,70^ The working principles of these sensors include mechanical nanocantilever-based, magnetic, electrochemical, and optical.^71^ Local physiological phenomena can be monitored using electrochemical-based single-analyte sensors, e.g., continuous glucose monitors (Figure 2D, E)^72,73^ and millimeter to centimeter-scale platforms that sense O_2_, H_2_, and CO_2_,^74^ as well as triethylamine and ammonia.^75^ Additionally, the functionalization of gold nanoparticles with single-stranded DNA fragments has been utilized for the detection of biomarkers and prognostic indicators for a variety of cancers.^71^ These include breast cancer indicators such as HER2, and BRCA1 fragments that have been developed with subzeptomolar detection limits.^76−78^ This could likely detect diseases such as cancer even before any visible signs appear, as detection is in the nanogram/mL scale, thereby increasing the chances of successful treatment.^79^

Dopamine detection, applied in the diagnosis of Parkinson’s, Huntington’s, and schizophrenia, has reached detection limits in the nmol/L scale in human serum using gold nanoparticle-based sensors.^80−82^ Multiple studies have focused on dopamine detection using carbon-based, graphene oxide, and carbon nanotubes, with micromolar-scale sensitivity in urine.^83,84^ Additionally, nanodiagnostics can increase the accuracy and swiftness of diagnostic tests through the passive uptake of nanoparticle imaging agents.^85^

Magnetic nanoparticles, specifically iron oxide-based nanoparticles, can upgrade imaging modalities such as Magnetic Resonance Imaging (MRI), forming highly detailed pictures for early diagnosis of diseases.^86^ This can be especially useful if portable imaging modalities have lower resolution than their gold-standard counterparts. Nanoparticle-based lateral flow assays, for example, can rapidly and accurately detect diseases and hormonal discrepancies (Figure 2F).^87−89^

By challenging the confines of diagnostics, nanotechnology-based tools play an important role in achieving miniature, sophisticated diagnostic tools. The thorough, personalized attention these devices can provide perfectly aligns with the principal goal of improving healthcare access and ameliorating treatment outcomes for all.

### Imaging

The recent advances in imaging technology have brought about an incredible transformation in the realm of diagnostic medicine. Portable and miniaturized devices like ultrasound (U/S) and MRI systems, now widely available, offer immediate, on-site diagnoses, which are particularly helpful in remote locations or emergency cases.^20,90^

U/S machines have been revolutionized thanks to the incorporation of novel U/S-on-chip technologies (Figure 2G).^91−93^ These point-of-care ultrasound (POCUS) devices include modern designs such as the Butterfly iQ (Butterfly Network, Inc., Guilford, CT) and Lumify (Philips, Amsterdam, Netherlands). Compatible with smartphones and tablets, POCUS devices allow healthcare professionals to get good resolution U/S images whenever necessary, and their applications extend far beyond emergency medicine and general practice to encompass rural healthcare and even disaster relief. Such devices are often supplemented with AI to determine and enhance image quality while also classifying the image.^93^ Additionally, piezoelectric-based wearable U/S patches have been developed with axial resolutions that allows for the detection of subcentimeter scale cysts in the breast,^94^ and even 48 h of continuous imaging of integral organs.^91^

Portable MRI Systems usually refer to low-field scanners (0.25–1T) designed to decrease manufacturing costs and increase access to devices.^95,96^ By continuing to address issues surrounding hardware and signal-to-noise ratio, portable MRI systems are on their way to being comparable with conventional MRI systems.^95,97^ For example, Swoop (Hyperfine, Guilford, CT)—a portable MRI system that is substantially smaller and lighter than its traditional counterparts and thus—allows for more flexible scanning of patients. With no need for a Faraday cage, the portable device can be wheeled right to the bedside for rapid diagnosis and treatment. This device successfully detected brain abnormalities in 97% of patients imaged while admitted to neuroscience intensive care units.^98^

The Pictor Plus Portable Ophthalmic Camera (Volk Optical Inc., Mentor, OH) has made it easier to capture high-resolution images of the retina for the diagnosis of conditions like diabetic retinopathy^99^ and glaucoma,^100^ particularly in locations where more sophisticated equipment is unavailable (Figure 2H).^101,102^ Additionally, Portable X-ray Devices, such as the HF120/60HPPWV PowerPlus (MinXray, Northbrook, IL), are now accessible to field hospitals, sports medicine, and veterinary practice, as they are highly portable, lightweight, and robust.^103,104^ An AI-driven image interpretation system has also been developed to complement the device use. In a study conducted in remote populations in Nigeria, an ultraportable X-ray device was used along with the AI interpretation to screen patients for tuberculosis.^105^ This approach saved 50% of the screenings needed for a correct diagnosis when compared to screening all symptomatic individuals.

Overall, portable imaging devices have proven to be indispensable in providing quick and local diagnoses, as well as offering better access to healthcare in remote or urgent cases. As time goes on, these tools are expected to continue making a significant contribution to improving patient care.

## Varying Clinical Decisions and Data Access

### Advanced Decision-Making Frameworks for Personalized Guidance

When acquiring medical data from a remote location, it is crucial to have self-sustaining decision-making systems that can analyze and interpret collected data. This is necessary to reduce the burden on healthcare systems. These systems should be capable of classifying whether a patient is at risk, determining the time frame, and suggesting whether they should seek out further medical attention from a healthcare provider.

Clinical Decision Support Systems (CDSSs) have existed since the 1970s, aiming to enhance medical decision-making through the use of general healthcare information, including patient data and clinical knowledge.^106,107^ Precision medicine aims to incorporate patient-specific medical data into clinical decision-making models to make patient-specific decisions.^108^ Without advanced CDSSs, such tasks are all but impossible.

CDSSs can be divided into knowledge and nonknowledge-based systems.^109^ While knowledge-based CDSSs are programmed to adhere to established medical knowledge, nonknowledge-based systems use AI or pattern recognition to guide clinical decisions.^107^ Both frameworks can be applied for personalized treatment guidance. CDSSs can perform administrative tasks and calculations of financial implications as well as inform diagnostic decisions, dose management, patient-drug matching, treatment plan optimization, etc.^110−112^ IBM’s Watson Assistant is an example of a general, natural language processing-based CDSS that integrates diagnostic capabilities into the clinical workflow, having learned the medical literature.^113,114^ Multiple pathology-specific systems have shown promise in aiding clinicians with diagnostic tasks in the fields of cardiology,^115^ dentistry,^116^ and endocrinology.^117^ As such, CDSSs not only speed up the clinical decision-making pipeline, relieving clinicians from dealing with certain “basic” decision-making tasks but have the potential to improve the quality of care by up to 5.8%.^118^ Additionally, CDSSs can ensure the integration of historical and real-time patient data for personalized decision-making.

#### Telemedicine and Remote Monitoring Tools

The world of healthcare is undergoing an exciting revolution with the emergence of telemedicine and remote monitoring solutions. These technologies are paving the way for every person to have access to quality care, transcending the geographical and temporal constraints that previously prevented access to critical care. Through real-time diagnosis, tracking, and management of patients from afar, medical expertise is now made remotely available to patients.

**Figure 3:**
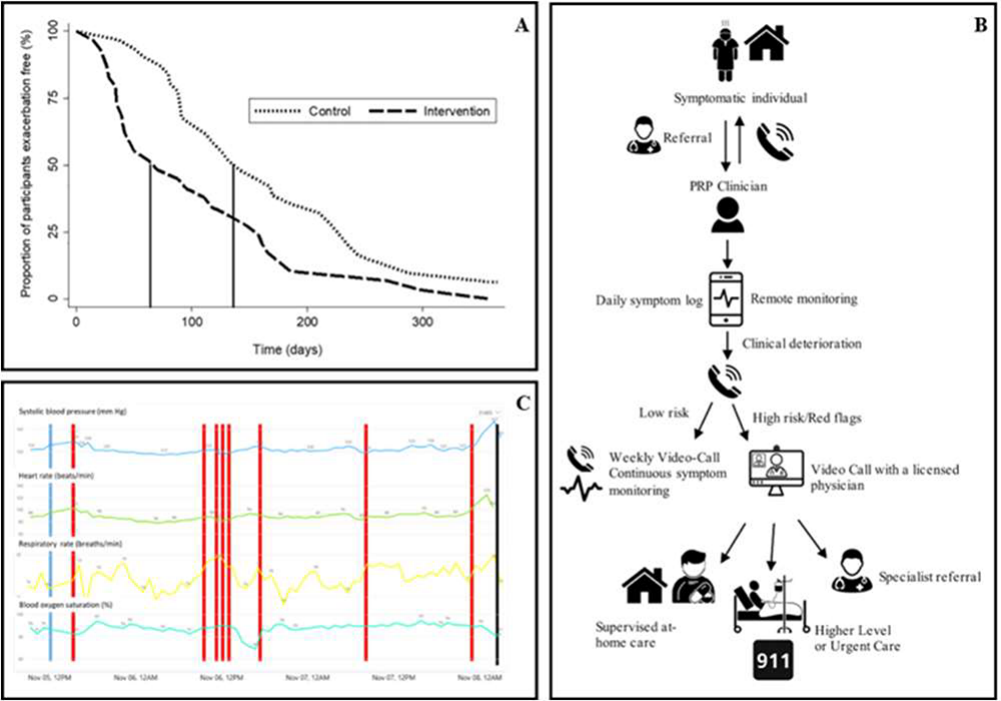
Empowering healthcare providers with RPM. (A) Time to detection of cystic fibrosis exacerbation using an application for symptom reporting. Reprinted with permission from Wood et al. Ref (122). Copyright 2019 Elsevier. (B) Workflow diagram of COVID-19 rpm. Reprinted with permission from Tabacof et al. Ref (140). Copyright 2021 Mary Ann Liebert, Inc. (C) Wearable Remote Patient Monitoring Device for the Early Detection of Patient Deterioration. Red lines indicate high-risk warnings while the terminal black line indicates the time of actual clinical deterioration. Reprinted with permission from Itelman et al. Ref (141). Copyright 2022 JMIR Publications Inc.

The beating heart of this transformation is Remote Patient Monitoring (RPM) Systems. RPM involves collecting data from a patient and wirelessly transmitting it a healthcare professional in another location.^119^ Of its numerous advantages, RPM enables continual monitoring of patients and real-time detection of diseases, leading to a potential reduction in the deterioration of illnesses and premature deaths.^120^ RPM programs can be employed by clinicians on several patient categories and are especially useful for patients suffering from chronic illnesses, such as diabetes, asthma, and cystic fibrosis, and neurological disorders, such as Parkinson’s disease and epilepsy.^121−123^ For example, a randomized control trial using a smartphone application for remote symptom monitoring for cystic fibrosis patients found a shorter time to detect disease exacerbation in the monitored group compared to the control (Figure 3A).^122^ In a 3-year study of pregnant women with type 1 diabetes to assess glycemic control while undergoing treatment, a telemedicine intervention involving daily treatment modification via a telephone call from a physician after examination of remotely acquired blood glucose levels showed statistically significant improvement in glycemic control for the study group that received the telemedicine intervention.^124,125^ These programs can allow patients to maintain certain elements of their daily lives while feeling secure in the knowledge that if their health deteriorates, a healthcare professional would be able to respond promptly.

The next step in this process is incorporating Virtual Consultation Platforms. These offer patients a personal consultation services with healthcare professionals who can be contacted via video conferencing or messaging. While there are some reservations as to the success of such consultation platforms due in large part to internet access and the chance of not picking up nonverbal cues, with the travel requirement removed, individuals living in remote areas or with mobility issues can access healthcare services easily.^126,127^ On top of that, Mobile Health Applications serve as personal health assistants, allowing patients to manage appointments, view medical records, connect with healthcare professionals, and get personalized health tips.^128,129^ To add to the medical expertise of these platforms, AI-Based Diagnostic Tools are also included. Using AI to analyze patient data, these tools can act as virtual consultants, assisting healthcare professionals in making speedy and accurate decisions in more complex cases.^130^

By uniting telemedicine and remote monitoring into healthcare delivery, the walls of traditional clinics are expanded to reach the patient, no matter their location.

#### Remote Access and Management of Patient Data for Healthcare Providers

Advancing technologies that continuously collect patient data in a noninvasive fashion requires cloud-based data access architectures that will enable healthcare providers to access the collected data remotely. This is essential in ensuring the continuity of care and collaborative partnerships among medical providers and is especially relevant in remote areas where medical professionals are unable to attend to patients physically. However, if appropriate access to collected data is provided, a high level of care for patients can still be maintained.^131^

The Health Level 7 (HL7) organization has incrementally developed standards for the exchange, sharing, and retrieval of electronic health records (EHRs) since 1987.^132^ The Fast Healthcare Interoperability Resources standard, published by HL7, is now widely accepted as the standard for healthcare data exchange framework built from modular components that can be assembled and used as part of mobile applications, communication with servers, and EHR sharing.^133^ These standards are essential in ensuring interoperability and widespread understanding of patient data. Multiple remote access systems have been built to comply with standards set by HL7.^134,135^

Mobile phones and tablets can be used for computation, data processing, and transfer of collected data, as well as for communication.^136,137^ Their widespread availability has drawn attention to mobile health (mHealth) under the electronic health (eHealth) and telemedicine umbrella. mHealth is useful in providing easy access to patient data in an efficient and time-conscious manner, allowing healthcare professionals to be more effective in clinical practice.^138^ Accessible patient data reduces information loss, shortens intervention times, allows for earlier detection of new health developments, reduces unnecessary testing and consultations, and promotes more effective decision-making, improving the overall standard of care even remotely.^139^

#### Empowering Healthcare Providers in Remote Patient Monitoring and Attention

Other than providing multiple benefits to patients, RPMs can serve as a load reduction tool for overwhelmed healthcare systems. For example, at the height of the COVID-19 pandemic in 2020, Mount Sinai Health System in New York launched an RPM program to provide care to symptomatic patients (Figure 3B).^140^ The program’s workflow allowed all symptomatic individuals tracked their symptoms and were subject to weekly video calls with a healthcare provider, while at-risk patients received close monitoring via a pulse oximeter and video calls with a physician. This allowed physicians to attend to high-risk patients efficiently.

Integrating RPM programs into existing healthcare frameworks, and providing patients with access to a range of diagnostic tools needed for the tracking of health parameters in MDCs, will ensure that healthcare providers keep their finger on the pulse regarding the latest changes in patient health status. This remote access to data will enable timely clinical intervention prior to patient deterioration (Figure 3C).^141^ It should be noted that such programs should be subjected to continual evaluation of their value to guarantee that the quality of care remains consistently good.

### Artificial Intelligence (AI)

**Figure 4:**
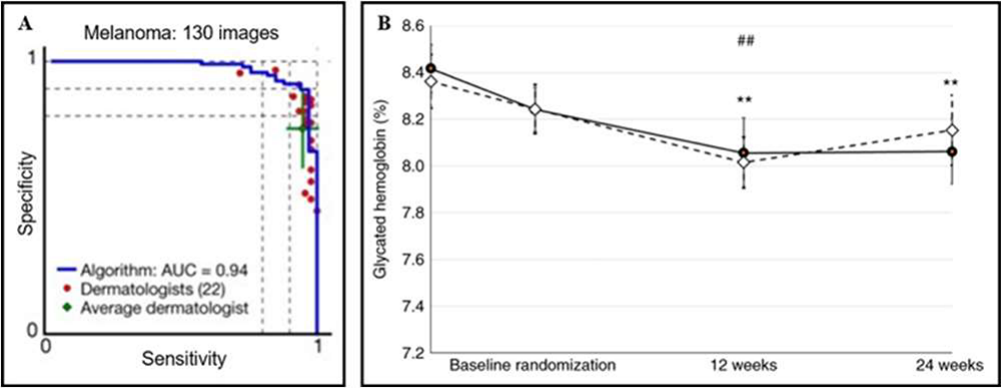
Implementing AI for real-time data assessment. (A) Skin cancer classification performance of a deep neural network and dermatologists. An AUC of 0.94 was achieved. Reprinted with permission from Esteva et al. Ref (15). Copyright 2017 Springer Nature. (B) Insulin dose optimization using an automated artificial intelligence-based decision support system (AI-DSS) in youths with type 1 diabetes. Filled circles represent the AI-DSS arm and the open diamonds represent the physician arm. Reprinted with permission from Nimri et al. Ref (17). Copyright 2020 Springer Nature.

AI has begun to, and will continue to, revolutionize healthcare. From the analysis of EHRs to scanning and deep learning model analysis of radiology or pathology images and classification of skin cancer with heightened accuracy and expediency (Figure 4A), personalized patient-treatment matching and dose optimization for diabetic patients (Figure 4B), and automation of appointment scheduling, AI capabilities are permeating the healthcare industry.^15,17,142^ The following instances illustrate AI’s transformative power in the healthcare sector.

AI-powered predictive analytics holds immense potential. AI algorithms can comb through large data sets, decipher patterns, and anticipate patient results, assisting healthcare practitioners in deciding on the most appropriate treatment options and preventive actions. For example, Yala et al. developed a deep learning-based model that assesses the breast cancer risk level of a patient by analyzing their full-field mammogram.^14^ The model achieved an AUC of 0.70, slightly higher than conventional risk prediction models. BioMind Technology (Beijing, China) has developed a U-NET-based model that successfully predicted early hematoma enlargement in patients with intracerebral hemorrhage, achieving sensitivity and specificity of 89.3% and 77.8%, respectively.^143^ These successes can help ensure timely clinical intervention.

AI is used in the diagnosis domain, where models aid clinicians in determining illnesses by rapidly inspecting medical images, pathology slides, and genomics with incredible accuracy. For instance, Rajpurkar et al. developed a convolutional neural network that can analyze chest X-rays and classify them into 14 different pathologies.^144^ The model’s performance level was on par with or better than experienced radiologists for 11 of the 14 tested pathologies. Esteva et al. developed a skin cancer classification model that outperformed board-certified dermatologists when classifying images of lesions into treat or not classes.^15^ The model achieved an AUC of 0.96 when classifying carcinomas. These models can save valuable clinician time while not compromising on diagnosis accuracy. This is particularly important when imaging is conducted remotely, away from experienced clinicians, as images can still be interpreted to a high level.

Another crucial application of AI lies in the form of virtual health assistants, driven forward by the advances in natural language processing. Text-based assistants have achieved commercial success but are limited to specific inputs for greater success rates.^145^ These AI-enabled assistants aid in patient healthcare by providing reminders to take medication, and as such, increasing medication adherence,^146−148^ monitoring symptoms, and offering health-related advice.^130,149^ AI can also analyze patient questions and direct them toward the correct healthcare resources, making professional healthcare guidance as attainable as making a phone call.^150,151^

#### Implementing AI for Real-Time Data Assessment

While certain AI-based tools have shown better accuracy than experienced physicians in disease classification tasks, real-time analysis of the collected data is essential for continuous monitoring devices.^152,153^ Real-time analysis of collected data is currently one of the most prominent bottlenecks in the field of healthcare big data.^154^ Reddy et al. simplified how AI can be used in healthcare systems by defining four fields: administrative tasks, CDSSs, patient monitoring, and clinician intervention.^155^ Each of these fields requires an understanding of the role AI could play and how relevant loads can be taken off healthcare providers while not only maintaining but improving the standard of care.

Currently, AI is used as a feature of CDSSs rather than as the sole decision-maker.^156^ Using AI for real-time data analysis collected by patient monitoring devices will aid in timely intervention, positively affecting clinical outcomes while also narrowing healthcare disparities between affluent and impoverished countries.^153^ However, the implementation of such systems is not yet widespread. In a study examining the use of AI-assisted CDSSs in the context of infectious diseases, 40% of systems were implemented in intensive care.^157^ In comparison, only 5% were used in the primary care setting, emphasizing the implementation gaps in the clinical workflow.

Several “black-box” algorithms, such as artificial neural networks, are used to analyze healthcare data. Yet, in the context of healthcare, for AI to be widely accepted and implemented, it must be “explainable”—the pathway to decisions or classifications of models must be traceable. Primarily, this would allow healthcare providers to understand the decisions being made, aiding the widespread implementation of AI in healthcare settings.^158^

## Implications and Challenges

As with any emerging technology, there is a pressing need to discuss the potential pitfalls of advances before major resources are invested in their development and deployment.

### A Harmonious Merging of Mobile Diagnostic Clinics into Patients’ Everyday Lives for Improved Accessibility and Comfort

Any device or platform that requires significant adjustments in the patient’s lifestyle or comfort level is more likely not to be utilized by the patient. Therefore, MDCs must be implemented in easily accessible locations for patients, such as community centers and malls. Devices incorporated as part of MDCs should be chosen carefully, prioritizing accessibility and adherence. Specifically, wearable devices must be comfortable, aesthetic, durable, easy to use, and exhibit health benefits to ensure high patient compliance. These implementation considerations require the selection of biocompatible materials for the skin-device interface. This is especially important in the design of the device’s electronics. Fabrication of stretchable and flexible electronics is a potential solution to question surrounding the comfort of devices.

Generally, material-based stretchable electronics have two components: an elastomer backbone and an electronic filler.^159^ The electronic filler is either metal- or carbon-based nanomaterial, including carbon nanotubes, carbon black, and graphene,^160−164^ or polymer-based conductors such as PEDOT:PSS^165^ and DPP-based polymers.^166^ The elastomer backbone can be chosen from a range of synthetic polymers such as polydimethylsiloxane (PDMS), polyethylene (PE), and poly(methyl methacrylate) (PMMA).^167−170^ This flexible backbone provides comfort for the wearer, increasing the device’s suitability for everyday use.

On the patient end, the integration of MDCs must be as seamless as possible. Patients should still feel cared for, respected, and not isolated, in addition to still receiving treatment that is at least on par with, if not better than, frontal, physician-only attention.

### Ethical aspects and data privacy worries

As health tech begins to realize the vision of MDCs, which will inevitably require distributing personal health data through cloud-assisted computing systems, understandable concerns arise involving medical data privacy and security. Data security involves measures that protect from unauthorized access, while healthcare data privacy involves regulations and technologies employed to protect sensitive patient medical records and protected health information (PHI).^171^ Data privacy aims to ensure that PHI remains accessible to healthcare professionals yet protected from ill-intentioned third parties and hackers. For example, in the US, the Health Insurance Portability and Accountability Act (HIPAA) of 1996 details national standards to protect patient health records and ensure they are not shared without the patient’s consent.^172^ Additional guidelines were published by the US National Institute of Standards and Technology (NIST).^173^ HIPAA is mirrored by the General Data Protection Regulation (GDPR) (Regulation (EU) 2016/679) in the European Union (EU) and the Data Protection Act in the UK, a supplement to the GDPR.^174,175^ Any medical device, healthcare provider, or company that deals with PHI must process and transmit the data it collects in compliance with these standards.

In a report published by the U.S. Department of Health and Human Services Office for Civil Rights detailing HIPAA breaches, it was estimated that 64,180 breaches affected approximately 37.5 million individuals in 2021.^176^ In 2022, it was estimated that 71.4% of medical record breaches were a result of hacking or IT incidents.^177^ As RPM platforms are conceptualized, and as such, more medical data about patients is collected, the dangers and potential to cause serious harm will continue to increase, creating not only financial losses for healthcare organizations through inevitable class action lawsuits and breach rectification but potentially serious health implications.

Multiple approaches to handling PHI have been proposed and are being utilized by healthcare providers and health tech companies. These approaches aim to combat breaches in both the data security and privacy planes. The main technologies used are authentication, encryption, data masking, access control, and monitoring and auditing.^171,178−189^ Additionally, methods for ensuring data privacy involve deidentification, hybrid execution models, and identity-based anonymization.^190^ Specifically, the blockchain, as detailed by Nakamoto,^191^ offers a potential solution to secure information transfer and access by providing a digitized, public, and distributed ledger. Multiple blockchain-based healthcare-oriented systems have been proposed, many utilizing smart contracts to automate data transaction protocols.^192−196^ All transactions of data are recorded, timestamped, and stored as a new block, which is appended to the existing blockchain, while the transaction is simultaneously verified and approved by all other users. As such, there is a detailed, public, and decentralized record of all parties with access to any patient record. By ensuring the secure transfer of files and storing pointers to the location of stored data in a decentralized database, patients can remain in control of their medical data while still providing a framework for real-time monitoring and clinical decision-making.^197^

While AI is revolutionizing healthcare, it is not without its issues—which will need to be addressed. According to studies, the prediction accuracy of ML algorithms varies by gender, race, and socioeconomic factors, magnifying preexisting biases.^198−200^ Additionally, AI-enabled systems are a “technical black box” in nature, which inhibit complete transparency of how clinical decisions are made. While progress is being made on the interpretability of ML algorithms, an opaque nature to how algorithms “decide” still exists, especially in the context of deep-learning-based neural networks. Explainable AI (XAI) refers to attempts at increasing AI decision-making transparency, which is especially important in the clinical setting.^201^ By providing explanations for classification outcomes, XAI can help increase trust in clinical decision-making models and deem them just and ethical. XAI is also essential to comply with the GDPR, which states that “a data subject [has the right]...to obtain an explanation of the decision reached.”^175,202^

### Implementation Problems in Healthcare Systems and Regulatory Frameworks

Implementing new medical procedures and technologies in the healthcare sector is a notoriously challenging task. Effective implementation of a novel device or concept requires a systematic, stepwise approach supported by evidence and analysis of the innovation while ensuring sustainable user growth and continuous evaluation of processes.^203^ Moreover, achieving widespread acceptance is difficult, especially in a field overflowing with contributors with different ideologies. As such, implementation processes must be detailed and mapped in advance, yet dynamic, to adjust for feedback from end-point users and medical caregivers alike.

Furthermore, regulatory frameworks are not universal and require market-differential solutions, which can complicate the global implementation and integration of medical devices into national healthcare systems. The Food and Drug Association (FDA) oversees the regulatory framework in the US, while devices deployed in the European Economic Area (EEA) require the undergoing of conformity assessment by notified bodies designated by EU countries under the European Medical Device Regulation (EU MDR).^204^ While EEA legislation, in force since 2018, has bridged certain regulatory differences between the E.U. and the U.S., some different regulatory standards still hinder global implementation.

Medical devices in the U.S. and E.U. are categorized according to risk level. These classifications determine the certification a device must have to be approved for use. Regardless of class, all devices must comply with general safety and performance standards, documentation, and postmarket surveillance. Generally, the higher the device class, the more stringent the regulatory proceedings. In the U.S., wearable devices require submission of either a 510(k), de novo, or premarket approval (PMA).^205^ These submissions necessitate either safety and efficacy comparisons to a predicate device, in the case of a 510(k) submission, or PMA or de novo if no predicate exists. Under the EU MDR, noninvasive, wearable devices with a measurement function are assigned “Class Im” as they pose low/medium risk.^204^ Such devices require regulation through a notified body before affixation of the CE marking and market approval.

The regulatory differences between markets make a universal approach to MDCs challenging. As such, the setup of MDCs between regions will likely be differently impacted by governing body regulations and existing healthcare frameworks.

#### Assuring Compatibility and Integration with Existing Healthcare Infrastructure

Healthcare systems have been molded into their current form for hundreds of years. For MDCs to be implemented, they must be able to sufficiently integrate within the setup of existing healthcare systems without completely shattering the existing mold.

Healthcare providers, specifically physicians, must still have access to patient data, allowing them to intervene in clinical decision-making when necessary. Practitioners must feel comfortable relinquishing parts of their clinical decision-making responsibilities to AI-assisted systems. This transition of responsibility will come through extensive testing of proposed models in clinical settings as clinical support systems, with the hope that through proof of exceptional performance, clinicians will feel comfortable trusting their capabilities.

### Dealing with Probable Confinements and Perils Affiliated with Mobile Diagnostic Clinics

Realizing the MDCs vision will require undergoing regulatory procedures, which may require the investment of massive resources. This could be financially and logistically taxing, and, as such, resources will need to be well allocated and managed.

From an ethical standpoint, questions over responsibilities need to be answered specifically regarding AI-assisted clinical decision-making. While CDSSs are important to analyze copious amounts of collected data, physicians must be able to correct any decisions that contradict their expert judgment.^206^ For this reason, it is important to develop transparent and interpretable AI-based tools. Additionally, emphasis should be placed on reducing AI bias through responsible representation of minority groups within training data sets.

Extensive measures must be taken to ensure data security and privacy. A data breach would expose the PHI of millions of patients, with dangerous consequences. While advances have been made in reducing the vulnerability of healthcare devices to data breaches, more work is needed to address these issues in a way that enables the efficient transfer of data from MDCs in a secure way that does not compromise patient integrity and rights to privacy.

Continuous monitoring platforms may be psychologically taxing for patients, contributing to an increased need to minimize the number of false alarms devices may trigger. While this will lead to alarm fatigue, it may also develop a lack of trust in the system by both physicians and patients.

Lastly, conceptualizing methods for analyzing the big data collected from MDCs, such that they serve their role in reducing physician load, is a bottleneck that will need to be solved if their implementation into the clinical workflow is to be expected soon.

## Conclusions and Outlook

The anticipated goal of the healthcare systems is to increase accessibility to cutting-edge diagnostic devices and platforms. This can be achieved by miniaturizing conventional diagnostic devices so that they can be used outside of traditional healthcare facilities. By increasing access to these devices, it becomes easier to continually monitor health. While significant developments have pushed the needle in creating suitable technologies for the implementation of “Mobile Diagnostic Clinics” (MDCs). Further research must still be done to realize this vision. Notably, issues surrounding ethics and data privacy must be addressed before any solution is rolled out to the general public as part of modern and evolving healthcare systems. When these issues are resolved, one can look forward to transformed and smart healthcare tailored to the specific needs of the individual. By increasing access to advanced technologies, this clinical model will allow patients to receive real-time updates on the state of their health while being sure that any potential issues are detected at the earliest possible stage to enable timely clinical intervention, improving healthcare outcomes.

## Abbreviations Used

WHO: World Health Organization
AI: Artificial Intelligence
POC: Point-of-Care
ML: Machine Learning
MDC: Mobile Diagnostic Clinic
BP: Blood Pressure
ECG: Electrocardiogram
PPG: Photoplethysmogram
ABP: Ambulatory Blood Pressure
CGM: Continuous Glucose Monitor
POCT: Point-of-care Testing
LOC: Lab-on-Chip
PCR: Polymerase Chain Reaction
U/S: Ultrasound
MRI: Magnetic Resonance Imaging
POCUS: Point-of-care Ultrasound
CDSS: Clinical Decision Support System
RPM: Remote Patient Monitoring
EHR: Electronic Health Record
mHealth: Mobile Health
eHealth: Electronic Health
AUC: Area under the Receiver Operating Characteristic Curve
PHI: Protected Health Information
HIPAA: Health Insurance Portability and Accountability Act
NIST: National Institute of Standards and Technology
GDPR: General Data Protection Regulation
EU: European Union
XAI: Explainable Artificial Intelligence
FDA: Food and Drug Administration
EEA: European Economic Area
EU MDR: European Union Medical Device Regulation
PMA: Premarket Approval
